# Phillyrin Prevents Neuroinflammation-Induced Blood–Brain Barrier Damage Following Traumatic Brain Injury *via* Altering Microglial Polarization

**DOI:** 10.3389/fphar.2021.719823

**Published:** 2021-10-20

**Authors:** Qian Jiang, Ding Wei, Xuejun He, Chao Gan, Xiaobing Long, Huaqiu Zhang

**Affiliations:** ^1^ Department of Neurosurgery, Tongji Hospital, Tongji Medical College, Huazhong University of Science and Technology, Wuhan, China; ^2^ Department of Neurosurgery, Tianyou Hospital Affiliated to Wuhan University of Science & Technology, Wuhan, China; ^3^ Department of Emergency, Renmin Hospital of Wuhan University, Wuhan, China

**Keywords:** traumatic brain injury, phillyrin, microglia, PPARγ, neuroinflammation, blood–brain barrier

## Abstract

**Background:** Phillyrin (Phi) is the main polyphenolic compound found in *Forsythia suspensa*. Recent studies have revealed that Phi has potent antioxidative and anti-inflammatory effects. However, whether Phi could relieve blood–brain barrier (BBB) damage following traumatic brain injury (TBI) remains unknown.

**Materials and Methods:** Lipopolysaccharide (LPS) was used to activate primary microglia, which were then treated with different doses of Phi or the peroxisome proliferator–activated receptor-gamma (PPARγ) antagonist (GW9662). CCK-8 assay was used for evaluating cell viability, and the cytokines (including IL-1β, IL-6, TNFα, IL-4, IL-10, and TGFβ), microglial phenotypic markers (iNOS, COX2, and CD86 for “M1” polarization; Arg1, Ym1, and CD206 for “M2” polarization), PPARγ, and NF-κB were determined by RT-PCR, Western blot, or cellular immunofluorescence. Primary cultured mouse brain microvascular endothelial cells (BMECs) were stimulated by the condition medium (CM) from microglia. The cell viability, angiogenesis, and tight junction of BMECs were determined *via* CCK-8 assay, tube formation assay, and Western blot (for detecting MMP3, MMP9, ZO1, claudin-5, and occludin). Furthermore, the mouse TBI model was constructed and treated with Phi and/or GW9662. The BBB integrity was evaluated by H&E staining, Evans blue staining, and tissue immunofluorescence.

**Results:** Phi markedly restrained the pro-inflammatory (“M1” state) cytokines and promoted anti-inflammatory (“M2” polarization) cytokines in LPS-mediated microglia. Phi mitigated “M1” polarization and promoted “M2” polarization of microglia *via* enhancing PPARγ and inhibiting the NF-κB pathway. The PPARγ antagonist GW9662 significantly repressed Phi-mediated anti-inflammatory effects. Meanwhile, Phi enhanced the viability, tube formation ability, and cell junction of BMECs. In the TBI mouse model, Phi promoted “M2” polarization, whereas it repressed the “M1” polarization of microglia. In addition, Phi reduced TBI-mediated BBB damage. However, the protective effects of Phi were reversed mainly by GW9662 treatment.

**Conclusion:** Phi prevents BBB damage *via* inhibiting the neuroinflammation of microglia through the PPARγ/NF-κB pathway, which provides a potential therapeutic drug against TBI.

## Introduction

Traumatic brain injury (TBI) often causes severe neurological impairment, which brings a heavy financial burden to the family and society (Rosenfeld et al., 2012). Emerging studies have shown that secondary inflammatory reactions following TBI exacerbate the damage (Corps et al., 2015; Corrigan et al., 2016). Damage-associated molecular patterns (DAMPs), such as high mobility group box protein 1 (HMGB1) and other cytokines, are released from the injured neurons or other injured cells, and then induce inflammation *via* direct effects on the downstream events associated with toll-like receptors (TLRs) (Tian et al., 2017; Paudel et al., 2018). TLR signal activation leads to blood–brain barrier (BBB) permeability, brain edema, and inflammatory reactions, thus aggravating brain damage.

The primary immune cells of the central nervous system (CNS), microglia, have a pivotal function in both the injury and protective processes following TBI (Izzy et al., 2019). Whether microglia exert a neuroprotective or damage-promotive effect depends on the stage of the disease and type of microglia activated in that particular stage of the disease (Tiwari et al., 2014). Several factors are involved in mediating the transformation of resting microglia (also called “M0” type) to the active M1 and M2 states (Kumar et al., 2016). For example, HMGB1, as an endogenous protein, becomes upregulated in neural and immune cells following TBI and activates the downstream MyD88/NF-κB pathway signaling of TLRs at the microglial cell surface, thus deriving “M1” microglia polarization (Xiong et al., 2016; Jassam et al., 2017; Gao et al., 2018). On the contrary, several cytokines, such as IL-4 and IL-10, and low-density lipoprotein receptor–related protein-1 (LRP1), could enhance the “M2” polarization of microglia following acute brain injury and improve neurological injury (Peng et al., 2019; Chen et al., 2020; Pu et al., 2021). The “M1” microglia release overexpressed pro-inflammatory cytokines (such as IL-1β, TNF-α, and IL-6), which result in further neuronal damage. In contrast, M2-type microglia release IL-4, IL-10, TGF-β, and other factors which can promote the repair of brain damage (Loane and Kumar, 2016). Activating the peroxisome proliferator–activated receptor-gamma (PPARγ) by rosiglitazone improved the neurological function and axonal injury *via* transforming the “M2” polarization of microglia (Wen et al., 2018; Liu et al., 2016). Thus, it is conceivable to modulate the formation of M1/M2 microglia, thereby reducing further inflammatory damage from taking place (Ni et al., 2019; Liu et al., 2021).

*Forsythia suspensa* (Thunb.) Vahl, also known as Lianqiao in Chinese traditional medicine, is often used in clinics for its extensive pharmacological activities (Gong et al., 2021). For example, the Forsythiae Fructus water extract has a hepatoprotective effect against carbon tetrachloride–induced liver fibrosis in mice (Zhang et al., 2018). In lipopolysaccharide (LPS)-induced acute lung injury, Forsythoside A (FA) (the active constituent of *Forsythia suspensa*) relieves inflammatory cytokine expression and prevents the abnormal adhesion and migration of monocytes to type II alveolar epithelial cells *via* enhancing miR-124 (Lu et al., 2020). As for phillyrin (Phi), another polyphenolic compound extracted from the leaves of *F. suspensa*, it has been found to have potent antioxidative and anti-inflammatory effects by activating the Nrf2 pathway or suppressing the NF-κB and MAPK signaling pathways (Du et al., 2020; Zhang et al., 2020). Interestingly, our study group found that Phi ameliorates neuronal apoptosis, cerebral edema, and microglia-mediated neuroinflammation following TBI (Jiang et al., 2020). However, the underlying mechanism of Phi on microglia polarization and BBB damage following TBI remains to be explored.

LPS-induced inflammation has been proposed as an *in vitro* model for several neurodegenerative disorders. For instance, C6 glial cells were treated with LPS to induce an *in vitro* model of neuropathic pain (Sharma et al., 2018). LPS is also used for inducing astrogliosis, a key contributor to many neurological disorders (Fernandes et al., 2018). Herein, we aim to study the role of Phi on microglia-mediated neuroinflammation and BBB damage in TBI. An *in vitro* model of microglial activation was induced by LPS, and an *in vivo* model on mice induced by CCI was applied. In addition, the condition medium of microglia was treated with BMECs. We found that the LPS or TBI insult promoted the pro-inflammatory reactions of microglia. At the same time, Phi exerted anti-inflammatory effects on microglia *via* promoting the “M2” polarization of microglia, and mitigated BMEC injury and integrity violation. In addition to that, Phi significantly inhibited the NF-κB pathway and promoted PPARγ expression. Therefore, we supposed that Phi relieves BMEC damage by altering the microglial polarization state through the PPARγ/NF-κB pathway.

## Materials and Methods

### Animals and Experimental Grouping

Fifty male and fifty female adult C57BL/10ScNJ mice (8 to 10 weeks old) weighing 20–22 g were obtained from the Animal Center of Tongji Medical College of Huazhong University of Science and Technology. Those mice were fed under specific pathogen-free (SPF) conditions and had access to a standard diet. When the mice were accustomed to the living environment, they were randomly divided into four groups: the Sham group (*n* = 20), TBI group (*n* = 20), TBI+Phi group (*n* = 20), and TBI+Phi+GW9662 group (*n* = 20). DMSO was used for dissolving Phi and GW9662 (Sigma-Aldrich, St Louis, MO, United States), which were diluted with 0.9% saline. Phi (10 mg/kg) and/or GW9662 (1 μmol/kg) was given immediately 1 h before surgery and after that daily for seven days by intraperitoneal injection as referred to in a previous study (Zhong et al., 2013; Donovan et al., 2015; Yang et al., 2017). The same volume of the solvent was given to the mice in the TBI or Sham groups.

### TBI Modeling

The mice received anesthesia by the administration of chloral hydrate [400 mg/kg body weight, intraperitoneally (i.p.)]. The TBI model induced by controlled cortical impact (CCI) was conducted as previously described (Yao et al., 2017). After surgery, the mice were kept in a warm environment before they woke up. The experimental protocols used in the present study, including all the surgical procedures and animal uses, were approved by Huazhong University of Science and Technology Committee for the Care of Animals (Wuhan, China) and followed the ARRIVE guidelines (Kilkenny et al., 2010).

### Evans Blue Staining

The Evans blue dye was used for the evaluation of BBB integrity (Shi et al., 2015). In brief, on the 7th day post-TBI, the mice were injected with Evans blue (2% in saline, 4 ml/kg, Sigma-Aldrich) *via* the tail vein. Two hours later, the mice were sacrificed and perfused with saline to remove the residual dye from the vessels. Next, the hemispheres of the mice were taken, and methanamide (Sigma-Aldrich) was used to incubate the tissues. After that, the percentage of the EB-stained brain volume was calculated (i.e., the ratio of the EB-stained volume of the ipsilateral hemisphere to the total volume of the contralateral hemisphere). In addition to that, the EB content of the hemispheres was tested by a trichloroacetic acid solution at 620 nm. The EB content was counted as intensity/mg of brain tissue.

### Primary Cell Culture

Primary microglia were obtained from C57BL/6 mice (1–2 days after birth), referring to our previous study (Long et al., 2020). The scattered cell mixtures were seeded in a culture flask with DMEM (supplemented with 10% FBS and 1% penicillin/streptomycin). The microglia were collected by shaking after 10 days of culturing. Cellular immunofluorescence was conducted to confirm the purity of microglia (labeled by Iba1).

Primary brain microvascular endothelial cells (BMECs) were obtained from 2-week-old C57BL/6 mice as described previously (Thomsen et al., 2015). In brief, the cerebral cortexes of the mice were collected before removing the meninges on the forebrains. Next, the tissues were cut into small pieces in an ice-cold DMEM. Meanwhile, a 5-ml pipette was used to dissociate the tissue patches further. Next, the tissue masses were digested in 10 ml DMEM that contained collagenase type 2 (1 mg/ml, Sigma), 300 μL DNase I (15 mg/ml, Sigma), and gentamycin (50 mg/ml, Sigma) on a shaker for 1.5 h at 37°C. After that, the cell pellet was separated by centrifugation in DMEM containing 20% bovine serum albumin (BSA) at 1,000 g for 20 min. The microvessel endothelial cell clusters were separated on a 33% continuous Percoll (Pharmacia, Uppsala, Sweden) gradient, collected, and washed twice in DMEM before plating on 35-mm plastic dishes coated with collagen type IV and fibronectin (both 0.1 mg/ml). The BMEC cultures were maintained in DMEM supplemented with 10% FBS, basic fibroblast growth factor (bFGF, Roche, Applied Sciences, Basel, Switzerland, 1.5 ng/ml), heparin (100 mg/ml), insulin (5 mg/ml), transferrin (5 mg/ml), sodium selenite (5 ng/ml) (insulin–transferrin–sodium selenite media supplement), gentamycin (50 mg/ml), and puromycin (4 mg/ml) at normal cell culture conditions. The endothelial cells were trypsinized when the cells reached 80% confluency.

### Cell Treatment

Primary microglia were cultured on 6-well plates with 5 × 10^5^ cells per well. Twelve h after seeding, the microglia were treated with LPS (10 ng/ml), Phi (0–40 μg/ml), or GW9662 (1 μM) for 4 h. Next, the culture medium was collected, and a new fresh complete medium was added. Centrifugation (1,000 rpm for 10 min) was used for removing the cell debris in the culture medium, which was then treated with primary BMECs seeded in 24-well plates (1 × 10^5^ cells per well) for 12 h. Then, the culture medium of BMECs was removed and supplemented with a new fresh culture medium. After another 24 h of culture, the culture medium of BMECs was collected for further experiments.

### Enzyme-Linked Immunosorbent Assay

After treating the condition medium of microglia, the culture supernatant from BMECs was collected. The level of VEGFA (Cat. No. 70-EK283/2-96, Elabscience, Shanghai, China) and EGF (Cat. No. EK0326, Wuhan, China) was determined using the ELISA kit according to the manufacturer’s protocols. The experiment was repeated five times.

### Western Blot

The microglia, BMECs, and brain tissues were collected, and the total proteins were separated by RIPA (Boyetime, Wuhan, China). The protein concentrations were determined using a BSA Kit (Boyetime, Wuhan, China). Next, the total proteins were isolated by SDS-PAGE and then transferred onto PVDF membranes. The membranes were blocked with 5% skimmed milk for 1 h at room temperature and then incubated with primary antibodies, including anti-MMP3 (1:1,000, ab52915, Abcam, United Kingdom), anti-MMP9 (1:1,000, ab228402, Abcam, United Kingdom), anti-iNOS (1:500, ab178945, Abcam, United Kingdom), anti-COX2 (1:1,500, ab179600, Abcam, United Kingdom), anti-CD86 (1:1,500, ab242142, Abcam, United Kingdom), anti-Arg1 (1:1,500, ab233548, Abcam, United Kingdom), anti-Ym1 (1:1,500, ab192029, Abcam, United Kingdom), anti-CD206 (1:1,500, Abcam, United Kingdom), anti-CD206 (1:1,500, Abcam, United Kingdom), anti-PPARγ (1:1,500, ab272718, Abcam, United Kingdom), anti–phospho-NK-κB (1:1,500, ab76302, Abcam, United Kingdom), anti–NK-κB (1:2,000, ab32536, Abcam, United Kingdom), anti-occludin (1:1,000, ab216327, Abcam, United Kingdom), anti-ZO1 (1:1,000, ab216880, Abcam, United Kingdom), and anti–claudin-5 (1:1,000, ab131259, Abcam, United Kingdom) at 4°C overnight. Next, the membranes were incubated with peroxidase-conjugated goat anti-mouse IgG secondary or peroxidase-conjugated goat anti-rabbit IgG (1:2,000, Abcam, United Kingdom) for 1 h at room temperature. Finally, the brands were exposed and photographed using a Gene Gnome exposure instrument. β-actin (1:2,000; Santa Cruz Biotechnology) was used as the internal reference of the other proteins. The experiment was repeated thrice.

### H&E Staining

The brain tissues of TBI mice on the 7th day were collected, fixed in 4% paraformaldehyde, embedded in paraffin, and dissected into sections with 10 μm thickness. Each section was then deparaffinized, hydrated, washed, and stained with hematoxylin–eosin (H&E) using a commercially purchased kit (Beyotime, Shanghai, China).

### Immunofluorescence Staining

The prepared brain sections or cells were permeabilized with Triton X-100 and blocked using goat serum. Then the sections or cells were incubated with primary antibodies including anti-occludin (1:100, Abcam, Cambridge,United Kingdom), anti–p-NF-κB (1:100, Abcam, United Kingdom), anti–Iba-1 (1:200, Abcam, Cambridge,United Kingdom), anti–ZO-1 (1:100, ab216880, Abcam, United Kingdom), anti-PPARγ (1:100, ab272718, Abcam, United Kingdom), anti-iNOS (1:200, ab178945, Abcam,United Kingdom), anti-Arg1 (1:200, ab233548, Abcam, United Kingdom), and anti–claudin-5 (1:150, ab131259, Abcam, United Kingdom) overnight at 4°C. On the next morning, the sections or cells were incubated with goat anti-mouse or goat anti-rabbit secondary antibodies conjugated to Alexa 488 or Alexa 647 (1:200; Abcam, Cambridge, United Kingdom) for 1 h at 37°C. The cell nuclei were stained with 4′,6-diamidino-2-phenylindole (DAPI) (Boyetime, Wuhan, China). After mounting, the immunofluorescent or immunohistochemical signals were observed under an Olympus microscope (Olympus, Tokyo, Japan). The positive cells were counted using Image J, with the researcher being blinded to the treatment conditions.

### Immunohistochemistry

The prepared brain sections were permeabilized with Triton X-100 and blocked with goat serum. Permeabilization and blocking were omitted for IgG immunostaining. The sections were incubated with primary antibodies of anti–caspase-3 (1:200, ab184787, Abcam, Cambridge,United Kingdom) overnight at 4°C. Then, the sections were incubated with the secondary antibody at room temperature for 2 h. After that, 3,3′-diaminobenzidine (DAB) chromogen was used to develop the peroxidase activity, and the nuclei were stained using 0.02% Mayer’s hematoxylin. Finally, the sections were observed under a camera-coupled bright-field microscope.

### Quantitative Real-Time PCR

Total RNA from the cells or tissues was isolated using TRIzol (Invitrogen, Carlsbad, CA, United States). Next, the total RNA was reverse-transcribed to cDNA with the PrimeScriptTM RT Reagent Kit (Thermo, United States) according to the manufacturer's instructions. The produced cDNA was amplified by quantitative real-time PCR on an ABI-Prism 7500 Real-Time PCR System (Applied Biosystems, Carlsbad, CA, United States) using SYBR Premix Ex TaqTM II (Takara). GAPDH was used as the internal control of the detected genes. The primer sequences used in this study were as follows: IL-1β, forward, 5′-ggc​tca​tct​ggg​atc​ctc​tc-3′, reverse, 5′-tca​tct​ttt​ggg​gtc​cgt​ca-3′; iNOS, forward, 5′-gtt​tga​cca​gag​gac​cca​ga-3′, reverse, 5′-gtg​agc​tgg​tag​gtt​cct​gt-3′; COX2, forward, 5′-ccc​caa​aca​cag​tgc​act​ac-3′, reverse, 5′-aga​ggt​tgg​aga​agg​ctt​cc-3′; CD86, forward, 5′-gca​cgt​cta​agc​aag​gtc​ac-3′, reverse, 5′-cat​atg​cca​cac​acc​atc​cg-3′; IL-6, forward, 5′-gga​gcc​cac​caa​gaa​cga​ta-3′, reverse, 5′-cag​gtc​tgt​tgg​gag​tgg​ta-3′; TNF-α, forward, 5′-gga​tta​tgg​ctc​agg​gtc​ca-3′, reverse, 5′-aca​ttc​gag​gct​cca​gtg​aa-3′; IL-4, forward, 5′-tgg​tgt​tct​tcg​ttg​ctg​tg-3′, reverse, 5′-acc​tgg​tag​aag​tga​tgc​cc-3′; IL-10, forward, 5′-aca​cct​tgg​tct​tgg​agc​tt-3′, reverse, 5′-tcg​ctt​tgt​aca​aca​gca​cc-3′; VEGFA, forward, 5′-gac​atc​ctc​ctc​cca​aca​ca-3′, reverse, 5′-att​acc​tgg​atg​ccg​caa​ac-3′, EGF, forward, 5′-cgg​aag​cag​cta​tca​aac​cc-3′, reverse, 5′-gag​aca​ggc​cag​cat​cta​ct-3′, TGF-β, forward, 5′-act​gct​tcc​cga​atg​tct​ga-3′, reverse, 5′-ttc​ctg​tag​aca​cac​cca​cc-3′; Arg1, forward, 5′-gct​ggg​aag​gaa​gaa​aag​gc-3′, reverse, 5′-tgc​cgt​gtt​cac​agt​act​ct-3′; Ym1, forward, 5′-ctc​aac​ctg​gac​tgg​cag​ta-3′, reverse, 5′-ctg​ctc​ctg​tgg​aag​tga​gt-3′; CD206, forward, 5′-aag​gaa​acc​atg​gac​aac​gc-3′, reverse, 5′-act​ttg​ctc​cca​tcc​atc​ca-3′; PPARγ, forward, 5′-aac​tcc​ctc​atg​gcc​att​ga-3′, reverse, 5′-gca​ttg​tga​gac​atc​ccc​ac-3′; and GAPDH, forward, 5′-aac​gac​ccc​ttc​att​gac​ct-3′, reverse, 5′-atg​tta​gtg​ggg​tct​cgc​tc-3′. The experiment was repeated thrice.

### Cell Counting Assay

Cell Counting Kit 8 (CCK-8) assay was used to evaluate the viability of microglia or BMECs using the CCK-8 kit (Cat. No. GK10001, Beyotime, Shanghai, China). Primary microglia or BMECs were cultured on 96-well plates with 5 × 10^3^ cells per well. Twelve hours after seeding, the microglia were treated with LPS (10 ng/ml), Phi (0–40 μg/ml), or GW9662 (1 μM) for 4 h. After that, 10 μl of the CCK-8 solution was added into each well, and the cells were incubated for 1 h at 37°C. Absorbance measurement at 450 nm was performed on the Thermo Scientific microplate reader. The value was used to calculate cell viability by setting the control as 100%. The experiment was repeated thrice.

### Tube Formation Assay

The capillary tube formation ability of BMECs was measured using Matrigel matrix (Cat. No. 354234; BD Biosciences). Matrigel matrix (50 μl in each well) was pre-coated on the 96-well plates 12 h before cell seeding, and the plates were put in the refrigerator at 4°C. After being treated with the condition medium from microglia for 12 h, the BMECs were collected and seeded (1 × 10^4^ cells/well) on the surface of the solidified Matrigel matrix and incubated for 12 h at 37°C. A light microscope (Olympus, Tokyo, Japan) was used for observing capillary tube formation. The length of the tubes was counted using ImageJ software (National Institutes of Health, Bethesda, MD, United States).

### Statistical Analysis

GraphPad 6.0 (GraphPad Software Inc., San Diego, CA, United States) was used for statistical analyses. All data are expressed as mean ± standard deviation (SD). Multiple comparisons were analyzed by one-way ANOVA followed by a *post hoc* Bonferroni correction. A non-paired *t*-test was used for the two groups’ data analysis. *p* < 0.05 was considered statistically significant.

## Results

### The Effects of Phi on the Polarization of Microglia Under LPS Stimulation Viability

**FIGURE 1:**
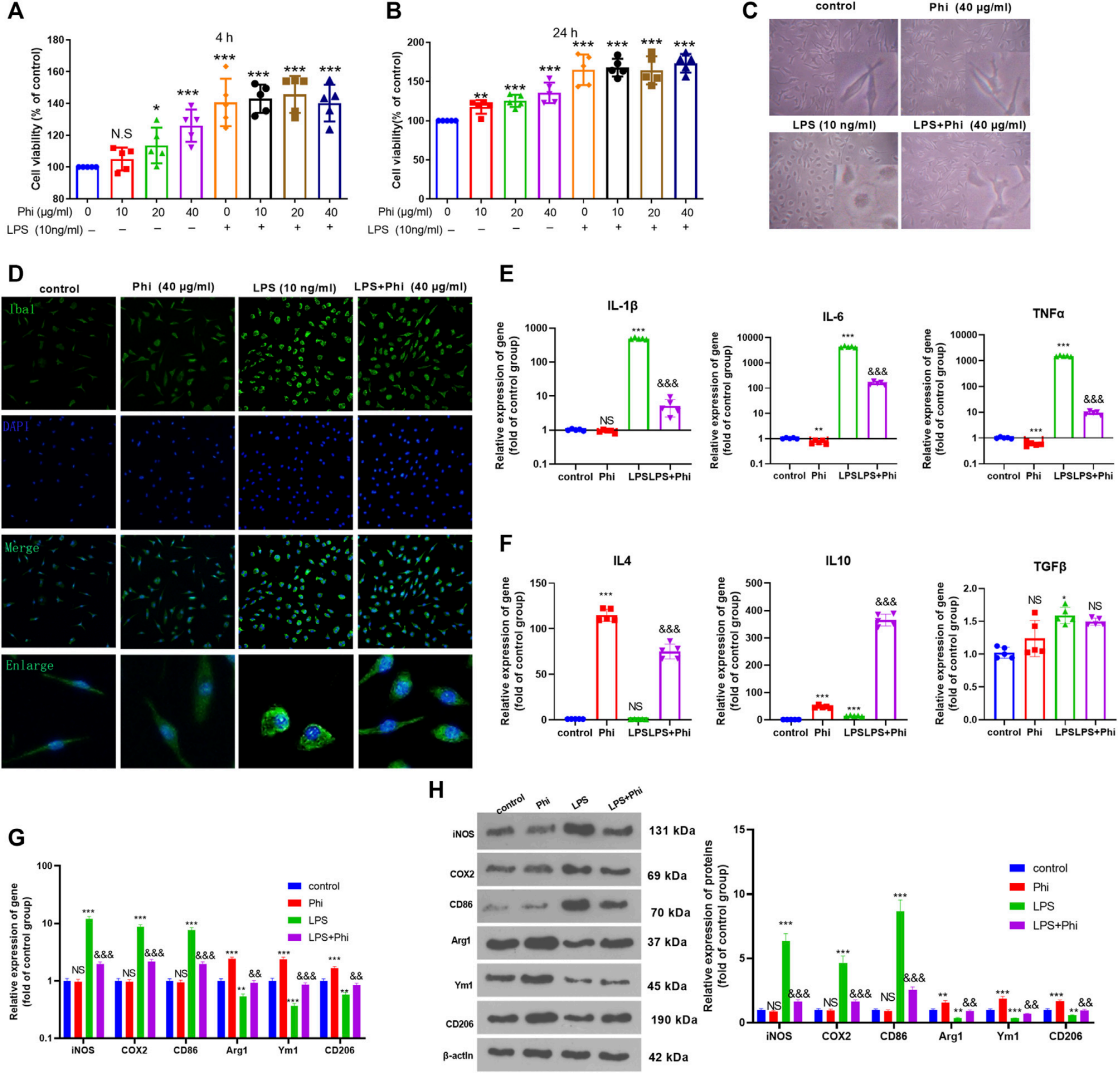
Effects of Phi on the viability and morphology of LPS-induced microglia. **(A**,**B)** Primary microglia were treated with LPS (10 ng/ml) and Phi (ranging from 10 to 40 μg/ml), or their combinations for 4 or 24 h. Then the viability of primary microglia was detected by the CCK-8 method. **(C)** Primary microglia were treated with LPS (10 ng/ml) and/or Phi (40 μg/ml) for 4 h. The morphological changes of microglia were recorded using a light Olympus microscope. **(D)** Cellular immunofluorescence was conducted to evaluate the microglial activation (labeled by Iba1). **(E**,**F)** RT-PCR was conducted to measure the “M1” markers of microglia, including IL-1β, IL-6, and TNFα, and “M2” markers, including IL-4, IL-10, and TGF-β. **(G**,**H)** RT-PCR or Western blot was conducted to detect the mRNA or protein levels of iNOS, COX2, CD86, Arg1, Ym1, and CD206 in microglia. The values are expressed as mean ±SD: NS *p* > 0.05, *p < 0.05, **p < 0.01, ***p < 0.001 vs. the control group; NS *p* > 0.05, && *p* < 0.01, &&&*p* < 0.001 vs. the LPS group. *n* = 5/group.

Primary cultured microglia were treated with different doses of Phi (0–40 μg/ml) or LPS (10 ng/ml). CCK-8 assay was performed to test the viability of microglia. The obtained data showed that all concentrations of Phi (ranging from 10 to 40 μg/ml) had no adverse effects on the viability of the microglial cells at 4 and 24 h with or without the insult of LPS (Figures 1A,B). Then, we detected the morphological changes of microglia using a light microscope and fluorescence microscope. The results showed that the microglia were in a state with long cell branches in the control group or Phi group. LPS stimulation transformed microglia into an active state with short cell branches and larger cell bodies. Nevertheless, Phi was inclined to decrease the ameba-like cells (Figures 1C,D). To investigate the effects of Phi on regulating the microglial inflammatory response, RT-PCR and Western blot were conducted. As the data showed, LPS treatment promoted the “M1” markers of microglia, including IL-1β, IL-6, TNFα, iNOS, COX2, and CD86 (compared with the control group, Figures 1E,G,H). The Phi treatment reduced IL-6 and TNFα levels, and promoted the expressions of “M2” markers, including IL-4, IL-10, Arg1, Ym1, and CD206 (compared with the control group, Figures 1E,G,H). Interestingly, the LPS+Phi group showed lower levels of IL-1β, IL-6, TNFα, iNOS, COX2, and CD86 but higher levels of IL-4, IL-10, Arg1, Ym1, and CD206 than the LPS group (Figures 1E–H). Collectively, Phi transformed the “M2” polarization of microglia.

### Phi Attenuated the Inflammatory Response of Microglia *via* Modulating the PPARγ/NF-κB Pathway

**FIGURE 2:**
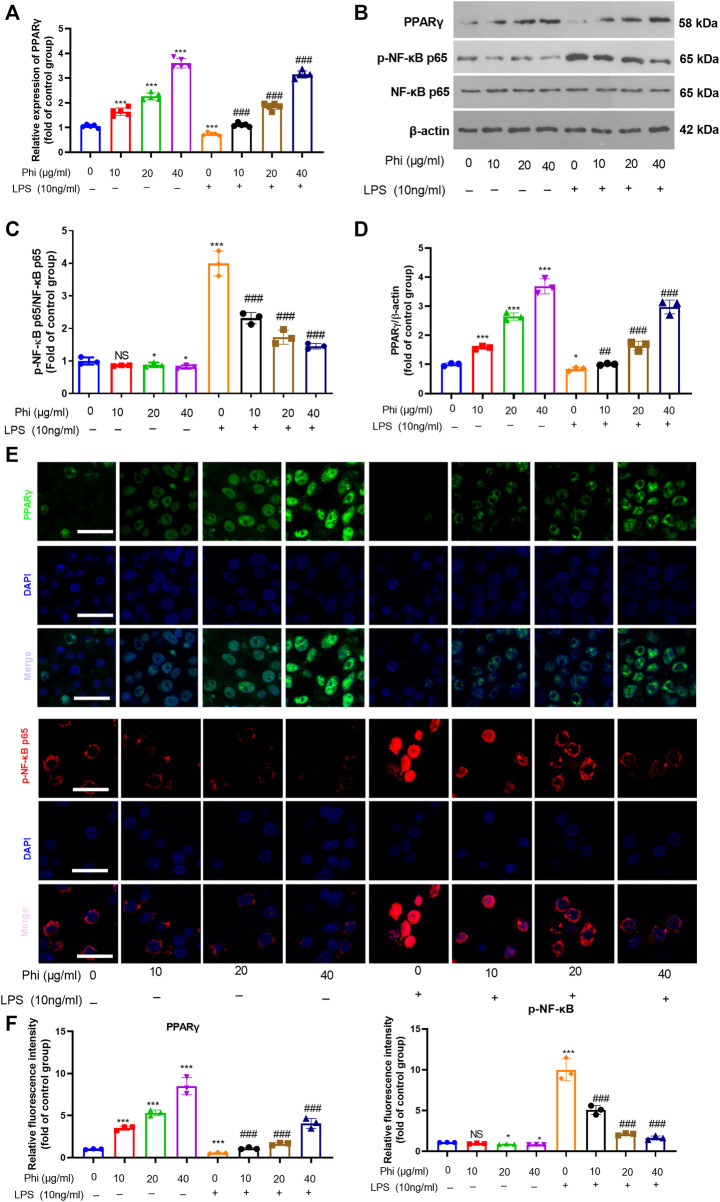
Effects of Phi on the PPARγ/NF-κB pathway in microglia under LPS stimulation. Microglia were treated with LPS (10 ng/ml) and/or Phi (40 μg/ml) for 4 h. **(A)** The PPARγ mRNA level in microglia was evaluated by RT-PCR. **(B**–**D)** Western blot was conducted to detect phospho-NK-κB p65, PPAR-γ in microglia. **(E**,**F)** Immunofluorescence was carried out to detect phospho-NK-κB p65, PPAR-γ in microglia. The relative fluorescence intensity of phospho-NK-κB p65 and PPAR-γ was analyzed by ImageJ. The values were expressed as mean ± SD. NS *p* > 0.05, *p < 0.05, **p < 0.01 ***p < 0.001 vs. the control group; ##*p* < 0.01, ###*p* < 0.001 vs. the LPS group. *n* = 5/group.

To further verify the mechanism of the anti-inflammatory effects of Phi *in vitro*, we first performed RT-PCR and Western blot to detect the PPARγ expression in microglia. As the data showed, Phi enhanced the mRNA and protein levels of PPARγ, whereas LPS reduced PPARγ mRNA and protein levels (compared with the control group, Figures 2A–D). However, Phi treatment on the LPS group promoted PPARγ mRNA and protein levels (compared with the LPS group, Figures 2A–D). Besides, the phosphorylated level of NF-κB was slightly reduced by Phi but promoted by LPS. However, the treatment of Phi suppressed the phosphorylated level of NF-κB (compared with the LPS group, Figures 2B–D). Next, PPARγ and phosphorylated NF-κB levels in microglia were detected by immunofluorescence. We found that Phi treatment promoted the PPARγ expression in the nuclei of microglia. After LPS treatment, the PPARγ expression was reduced, and phosphorylated NF-κB was increased and translocated into the nuclei. However, Phi treatment not only promoted the PPARγ expression but also reduced the nuclear translocation of NF-κB (Figures 2E,F). As a result, we believed that Phi might modulate the polarization of microglia *via* the PPARγ/NF-κB pathway.

### Inhibition of PPARγ Reversed Phi-Mediated “M2” Polarization of Microglia

**FIGURE 3:**
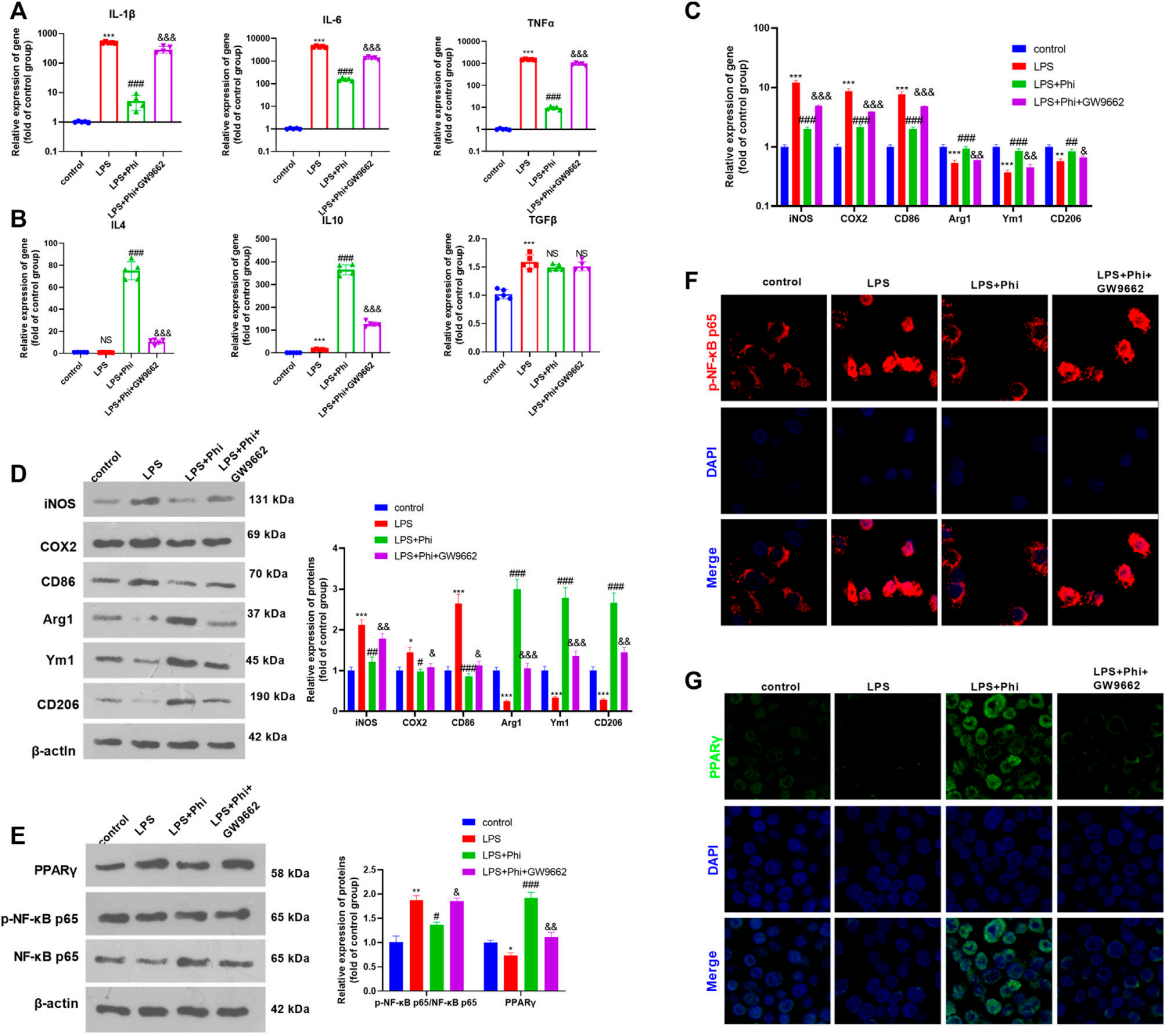
Phi inhibited the inflammatory response of LPS-activated microglia *via* the PPARγ signaling pathway. The microglia were treated with LPS (10 ng/ml) or phillyrin (40 μg/ml) or GW9662(1 μM) for 4 h. **(A**,**B)** RT-PCR was conducted to measure the “M1” markers of microglia, including IL-1β, IL-6, and TNFα, as well as “M2” markers, including IL-4, IL-10, and TGF-β. **(C**,**D)** RT-PCR or Western blot was conducted to detect the mRNA or protein levels of iNOS, COX2, CD86, Arg1, Ym1, and CD206 in microglia. **(E)** Western blot was conducted to measure phospho-NK-κB p65 and PPAR-γ in microglia. **(F**,**G)** Immunofluorescence was carried out to detect phospho-NK-κB p65, PPAR-γ in microglia. The values are expressed as mean ± SD. NS *p* > 0.05, *p < 0.05, **p < 0.01, ***p < 0.001 vs. the control group; NS *p* > 0.05, #*p* < 0.05, ##*p* < 0.01, ###*p* < 0.001 vs. the LPS group; NS *p* > 0.05, &*p* < 0.05, &&*p* < 0.01, &&&*p* < 0.001 vs. the LPS+Phi group. *n* = 5/group.

To verify the role of the PPARγ/NF-κB pathway on Phi-mediated transformation of microglial polarization, we treated microglia with GW9662 (1 μM), a PPARγ antagonist. We then treated microglia with Phi (40 μg/ml) or LPS (10 ng/ml). After that, RT-PCR and Western blot were conducted to determine the inflammatory reactions of microglia. As the data showed, GW9662 treatment promoted the expression of “M1” markers in microglia, including IL-1β, IL-6, TNFα, iNOS, COX2, and CD86, whereas it inhibited the expressions of “M2” markers, including IL-4, IL-10, Arg1, Ym1, and CD206 (compared with the LPS+Phi group, Figures 3A–D). We then conducted Western blot and cellular immunofluorescence to detect the PPARγ/NF-κB pathway expression. It was found that the addition of GW9662 in the LPS+Phi group reduced the PPARγ expression and promoted the phosphorylated NF-κB in the nuclei in microglia (Figures 3E–G). Therefore, Phi transformed the “M2” polarization of microglia dependently through modulating the PPARγ/NF-κB pathway.

### Phi Promoted BMEC Viability and Tube Formation Ability *via* PPARγ in Microglia

**FIGURE 4:**
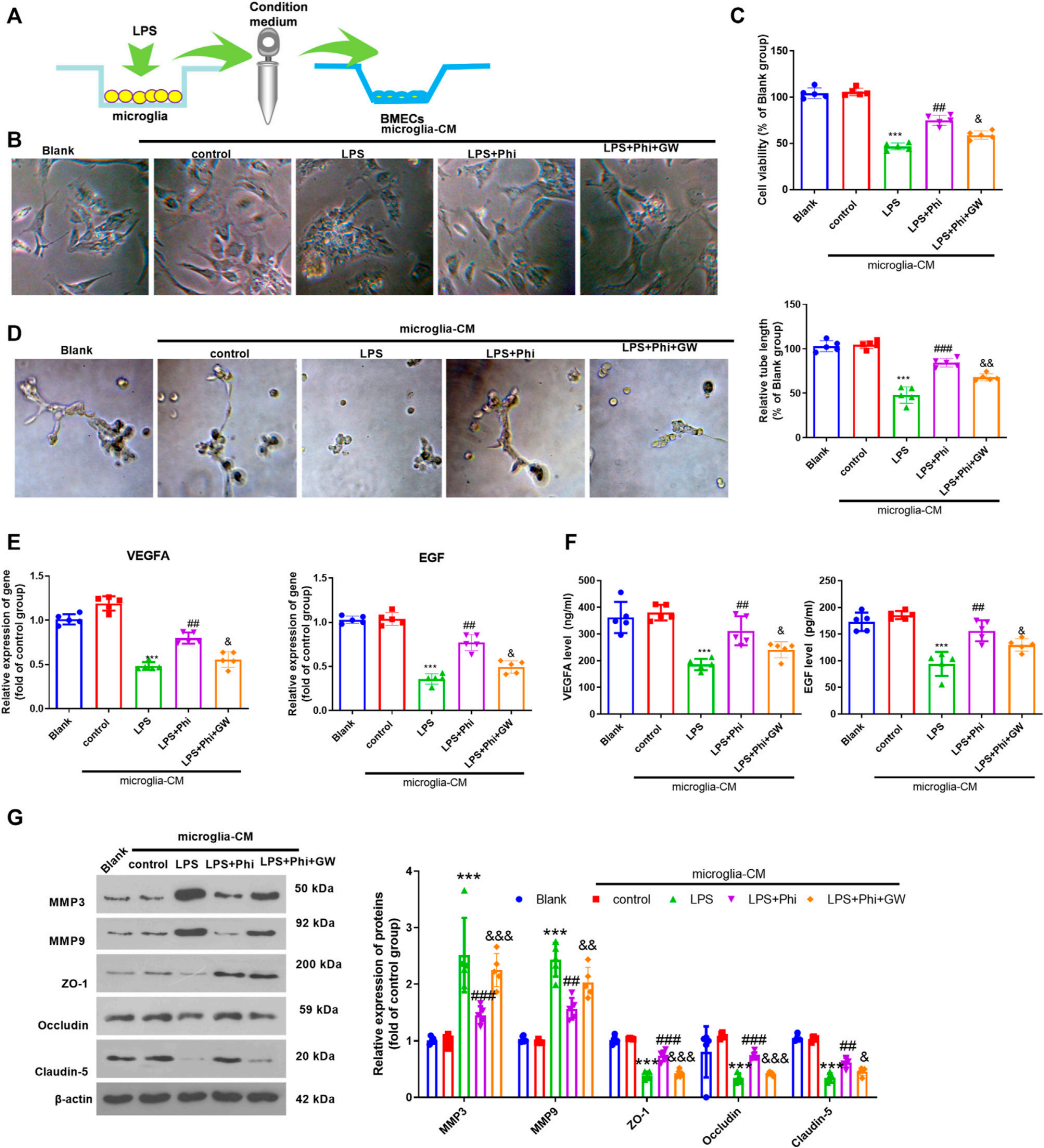
Phi promoted BMECs viability and tube formation ability via PPARγ in microglia. **(A)** Microglia were treated with LPS (10 ng/ml) or phillyrin (40 μg/ml) or GW9662(1 μM) for 4 h. BMECs were treated with the culture medium from microglia. **(B)** Morphological changes of BMECs were recorded by using a light Olympus microscope. **(C)** Cell viability of BMECs was detected by CCK-8 assay. **(D)** Tube formation assay was performed to evaluate the tube formation ability of BMECs. **(E**,**F)** RT-PCR and ELISA were conducted to measure VEGFA and EGF in BMECs or the culture medium. **(G)** Expressions of MMP3, MMP9, ZO-1, occludin, and claudin-5 in BMECs were evaluated via Western blot. The values are expressed as mean ± SD. ***p < 0.001 vs. the control-CM group; ##*p* < 0.01, ###*p* < 0.001 vs. the LPS-CM group; &*p* < 0.05, &&*p* < 0.01, &&&*p* < 0.001 vs. the LPS+Phi-CM group. *n* = 5/group.

We treated BMECs with the culture medium from microglia (Figure 4A). The cell viability of BMECs was observed using a light microscope and detected by CCK-8 assay. The results showed that when compared with the Blank group, the condition medium from microglia in the control group did not alter the viability of BMECs. The condition medium from LPS-treated microglia inhibited the viability of BMECs (compared with the microglia–CM control group, Figures 4B,C). However, Phi+LPS–treated microglia showed fewer inhibitive effects on the viability of BMECs, which were reversed by GW9662 treatment in the microglia (Figures 4B,C). Next, we performed a tube formation assay to evaluate the tube formation ability of BMECs. It was found that the condition medium from LPS-induced microglia significantly inhibited the tube formation ability of BMECs (compared with the control-CM group). Phi treatment in LPS-induced microglia promoted the tube formation ability of BMECs (compared with the LPS-CM group), whereas this effect was reversed with GW9662 treatment in microglia (compared with the LPS+Phi-CM group, Figure 4D). Next, we conducted RT-PCR and ELISA to measure VEGFA and EGF in BMECs or the culture medium. As the data showed, the condition medium from LPS-induced microglia repressed VEGFA and EGF expressions (compared with the control-CM group). The LPS+Phi–treated microglial condition medium resulted in enhanced expressions of VEGFA and EGF, which were reversed by GW9662 (compared with the LPS+Phi-CM group, Figures 4E,F). Furthermore, we evaluated the expressions of MMP3, MMP9, ZO-1, occludin, and claudin-5 in BMECs under the stimulation of the condition medium from microglia. We found that LPS-CM promoted MMP3 and MMP9, whereas it inhibited ZO-1, occludin, and claudin-5 in BMECs (compared with the control-CM group). However, the LPS+Phi-CM group reduced MMP3 and MMP9 levels, whereas it accelerated ZO-1, occludin, and claudin-5 expressions in BMECs (compared with the LPS-CM group). In addition, GW9662 treatment in LPS-mediated microglia had the opposite effect (Figure 4G). Hence, those results indicated that Phi attenuated microglia-mediated BMECs through PPARγ in microglia.

### Phi Promoted BMEC Viability and Tube Formation Ability *via* PPARγ in BMECs

**FIGURE 5:**
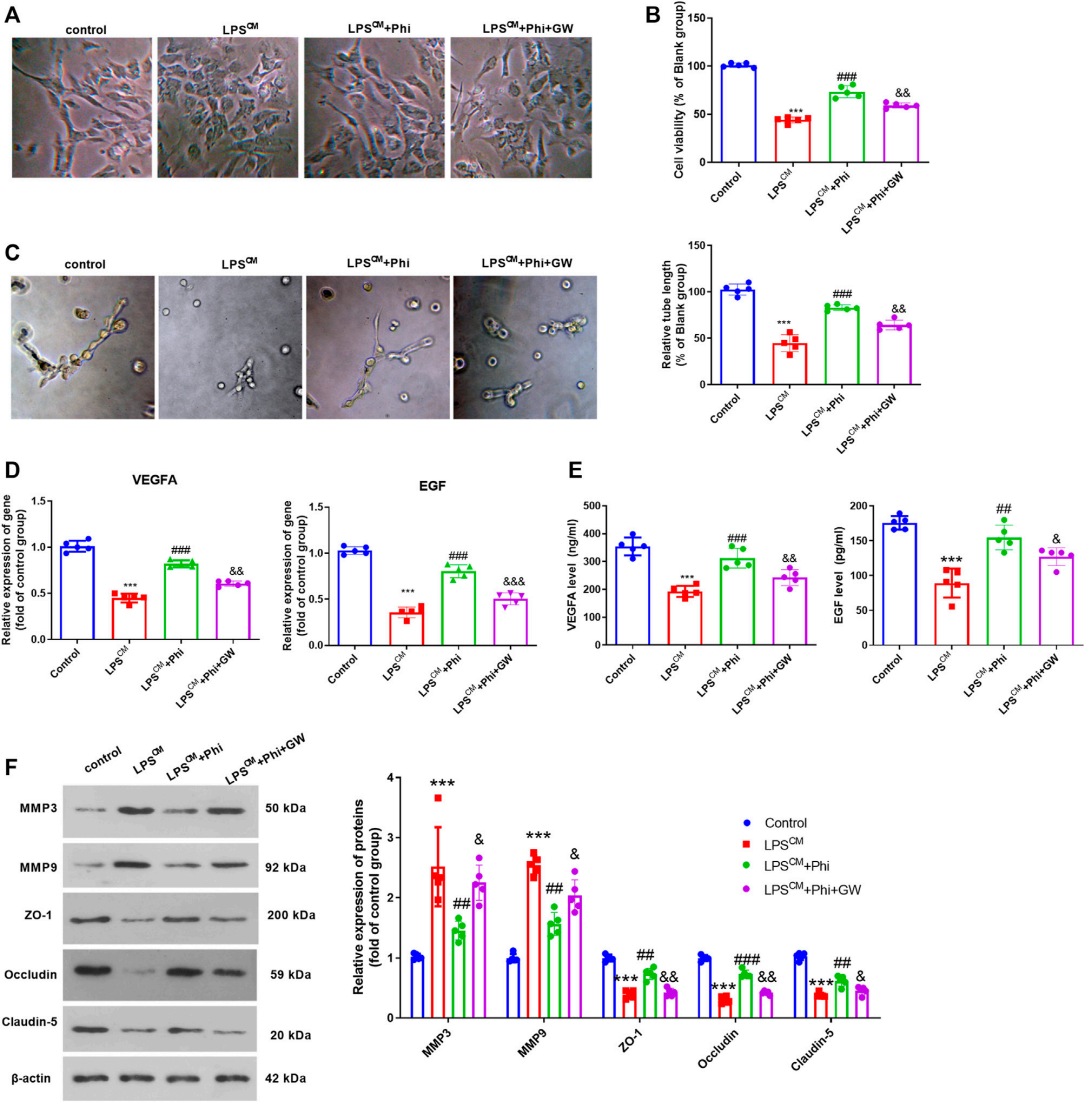
Phi promoted BMECs viability and tube formation ability via PPARγ in BMECs. BMECs were treated with the culture medium from LPS-mediated microglia, Phi (40 μg/ml) and/or GW9662. **(A)**Morphological changes of BMECs were recorded using a light Olympus microscope. **(B)** Cell viability of BMECs was detected by CCK-8 assay. **(C)** Tube formation assay was performed to evaluate the tube formation ability of BMECs. **(D**,**E)** RT-PCR and ELISA were conducted to measure VEGFA and EGF in BMECs or the culture medium. **(F)** Expressions of MMP3, MMP9, ZO-1, occludin, and claudin-5 in BMECs were evaluated via Western blot. The values are expressed as mean ± SD. ***p < 0.001 vs. the control group; ##*p* < 0.01, ###*p* < 0.001 vs. the LPS-CM group; &*p* < 0.05, &&*p* < 0.01, &&&*p* < 0.001 vs. the LPS-CM +Phi group. *n* = 5/group.

We treated BMECs with the culture medium from LPS-mediated microglia, Phi (40 μg/ml), and/or GW9662. The cell viability and tube formation ability of BMECs were repressed by LPS-CM stimulation. Phi treatment in BMECs significantly enhanced BMEC cell viability and tube formation ability (compared with the LPS-CM group), whereas GW9662 treatment mostly reversed Phi-mediated effects (compared with the LPS-CM+Phi group, Figures 5A–C). Additionally, we detected the alteration of VEGFA and EGF in BMECs or the culture medium. It was found that compared with the LPS-CM group, both VEGFA and EGF were promoted with Phi treatment. However, GW9662 addition inhibited VEGFA and EGF in BMECs (Figures 5D,E). In addition to this, we detected the expressions of MMP3, MMP9, ZO-1, occludin, and claudin-5 in BMECs. We found that Phi inhibited MMP3 and MMP9, whereas it promoted ZO-1, occludin, and claudin-5 in BMECs (compared with the LPS-CM group). However, the LPS-CM+Phi+GW9662 group had increased MMP3 and MMP9 levels and downregulated ZO-1, occludin, and claudin-5 expressions in BMECs (compared with the LPS-CM+Phi group, Figure 5F). Consequently, the abovementioned data suggested that Phi attenuated microglia-mediated BMEC damage *via* PPARγ.

### Phi Promoted “M2” Polarization of Microglia in the TBI Mouse Model

**FIGURE 6:**
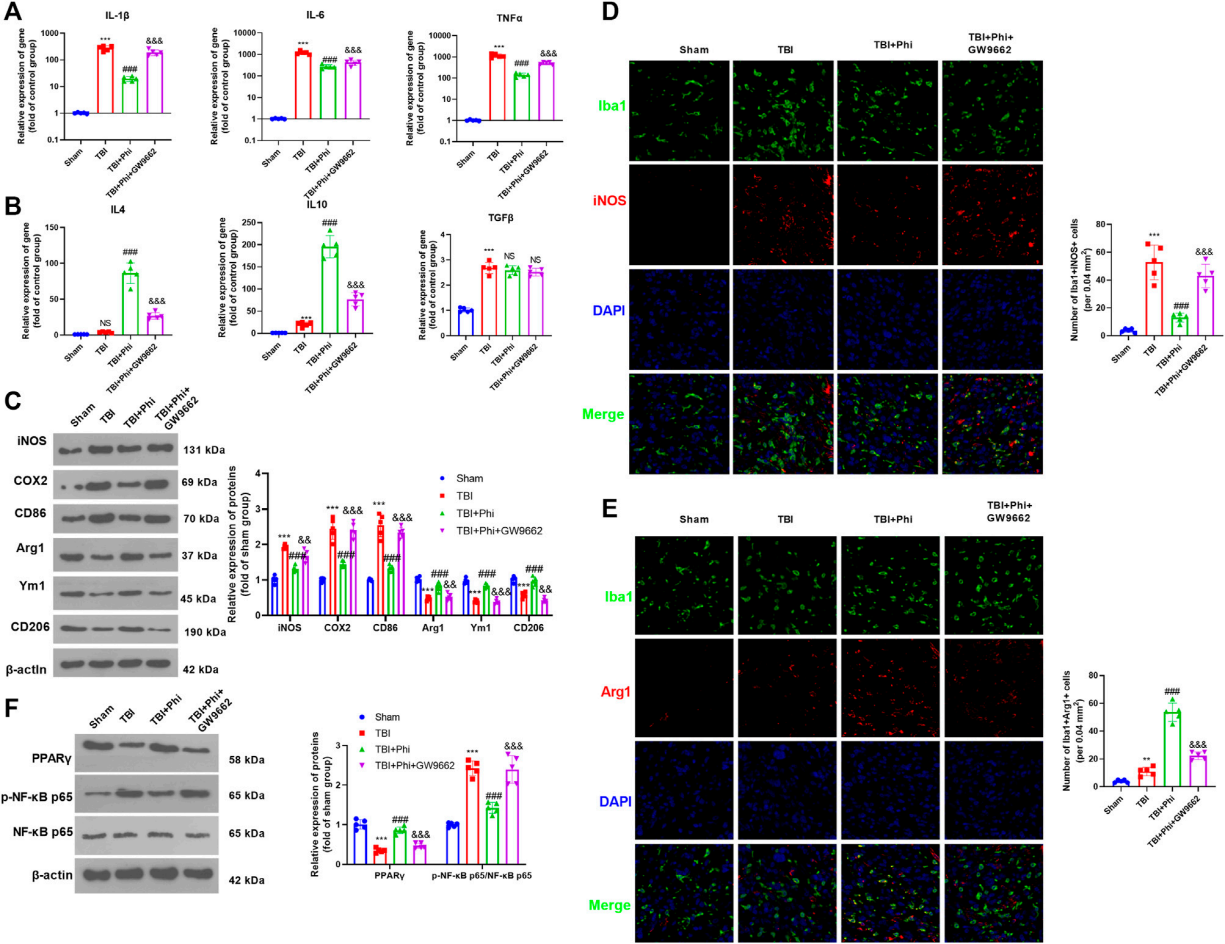
Phi inhibited the inflammatory response of TBI-activated microglia. The TBI mouse model was constructed. The mice subjected to TBI were treated with Phi (10 mg/kg) and, or GW9662 (1 μmol/kg) 1 h before surgery and thereafter daily for seven days by intraperitoneal injection. The same volume of the solvent was given for the mice in the TBI or sham group. **(A**,**B)** RT-PCR was conducted to measure IL-1β, IL-6, TNFα, IL-4, IL-10, and TGF-β in the brain lesions seven days after TBI. **(C)** Western blot was conducted to detect the protein levels of iNOS, COX2, CD86, Arg1, Ym1, and CD206 in TBI lesions seven days after TBI. **(D**,**E)** Immunofluorescence was used to detect Iba1^+^iNOS^+^ and Iba1^+^Arg1^+^microglia in the brain lesions seven days after TBI. **(F)** Western blot was conducted to measure phospho-NK-κB p65 and PPAR-γ in the brain lesions seven days after TBI. The values are expressed as mean ±SD. NS *p* > 0.05, ***p < 0.001 vs. the sham group; NS *p* > 0.05, ###*p* < 0.001 vs. the TBI group; NS *p* > 0.05, &&*p* < 0.01, &&&*p* < 0.001 vs. the TBI+Phi group. *n* = 5/group.

Our previous study had suggested that Phi relieved the neurological deficits of the TBI mice and inhibited neuron apoptosis in the brain lesions (Jiang et al., 2020). To further explore the mechanism of Phi on the polarization of microglia on the TBI mouse model, we first performed RT-PCR to detect the “M1/M2” polarization markers in the brain lesions. As the data showed, in the TBI group, the expressions of IL-1β, IL-6, TNFα, iNOS, COX2, and CD86 were all significantly upregulated compared with those of the sham group (Figures 6A,C). After the administration of Phi, IL-1β, IL-6, TNFα, iNOS, COX2, and CD86, the levels were reduced, while the “M2” markers, including IL-4, IL-10, Arg1, Ym1, and CD206, were all promoted compared with the TBI group (Figures 6B,C). Next, we conducted immunofluorescence to examine the polarization state of microglia. Our data showed that compared with the TBI group, the number of Iba1^+^iNOS^+^-labeled microglia was significantly increased. Phi treatment promoted the number of Iba1^+^Arg1^+^-labeled microglia, while it reduced Iba1^+^iNOS^+^-labeled microglia compared with the TBI group (Figures 6D,E). Furthermore, Western blot results showed that PPARγ was inhibited, and the p-NF-κB p65 level was promoted in the TBI group. By contrast, Phi promoted the PPARγ level and inhibited the p-NF-κB p65 level (compared with TBI group, Figure 6F). Therefore, we believed that Phi ameliorated the neuroinflammation of TBI mice *via* transforming the “M2” polarization of microglia.

### Phi Improved BBB Damage in the Brain Lesions of TBI Mice

**FIGURE 7:**
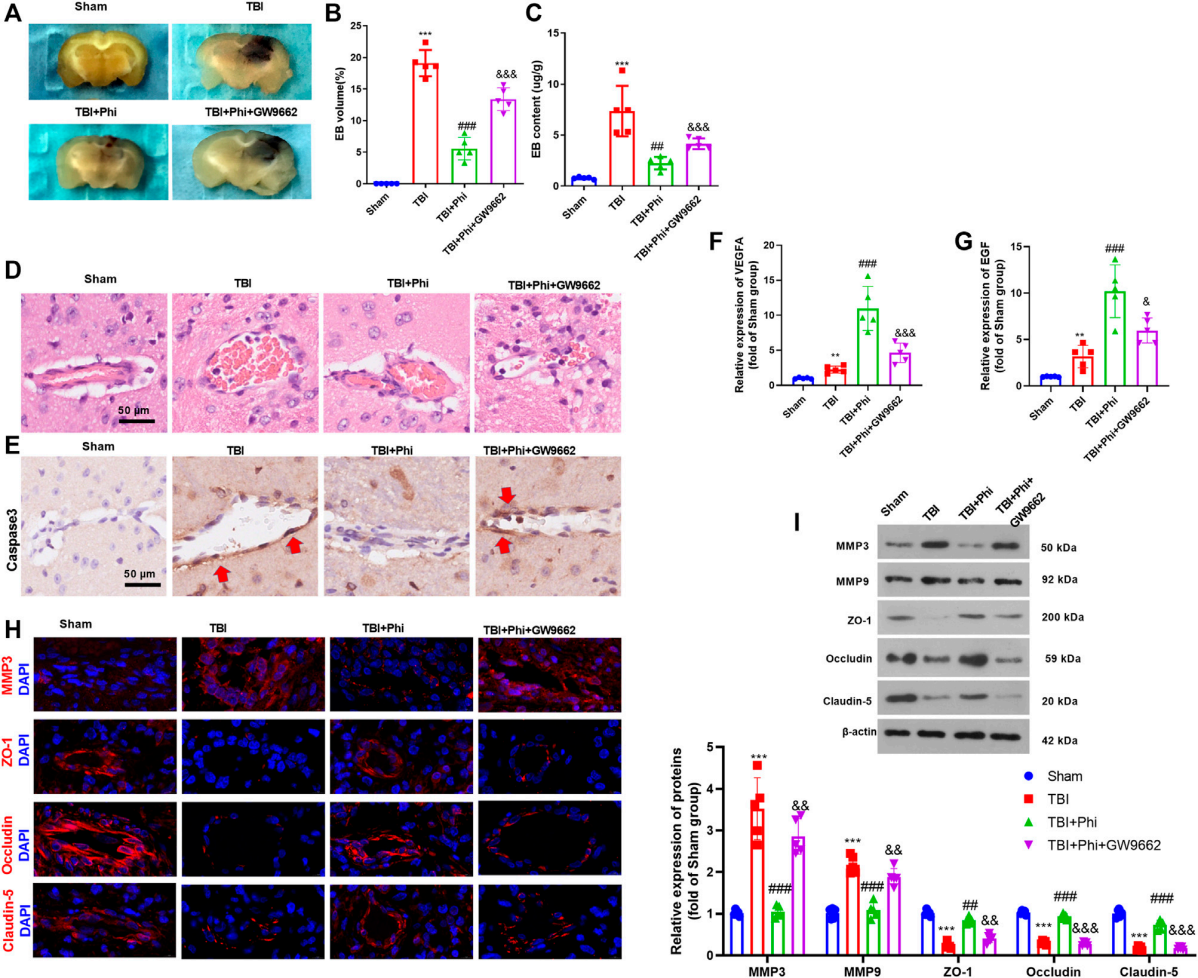
Phi mitigated BBB damage on TBI mice. The mice subjected to TBI were treated with Phi (10 mg/kg) and/or GW9662 (1 μmol/kg) 1 h before surgery and thereafter daily for seven days by intraperitoneal injection. The same volume of the solvent was given for the mice in the TBI or sham group. **(A**–**C)** BBB permeability was investigated by measuring the extravasation of Evans blue dye (*n* = 5). **(D**,**E)** HE and IHC (anti-caspase-3) were used to observe the integrity of cerebral microvessels. **(F**,**G)** RT-PCR was conducted to measure VEGFA and EGF in BMECs in the brain lesions. **(H)** Tissue immunofluorescence was conducted to detect MMP3, ZO-1, occludin, and claudin-5 in the brain lesions. **(I)** Western blot was used to detect the protein level of MMP3, MMP9, ZO-1, occludin, and claudin-5 in the brain lesions (*n* = 5). The values are expressed as mean ± SD. **p < 0.01, ***p < 0.001 vs. the sham group; ##*p* < 0.01, ###*p* < 0.001 vs. the TBI group; &&*p* < 0.01, &&&*p* < 0.001 vs. the TBI+Phi group. *n* = 5/group.

BBB permeability was investigated by measuring the extravasation of Evans blue dye. The results revealed that TBI caused a significant increase of Evans blue dye extravasation, which was then decreased under the treatment of Phi (vs. the TBI group, Figures 7A–C). The addition of GW9662 increased Evans blue dye extravasation (compared with the TBI+Phi group, Figures 7A–C). In addition to this, the results of the pathological examinations showed that the BBB integrity was significantly destroyed in the TBI brain lesions, and caspase-3–labeled apoptotic BMECs were increased (Figures 7D,E). However, Phi relieved BBB damage and reduced caspase-3–positive cells in the cerebral microvessels, and GW9662 partly reversed Phi-mediated effects (Figures 7D,E). RT-PCR was performed to detect VEGFA and EGF in the TBI lesions. It was found that TBI resulted in a downregulated level of VEGFA and EGF, and Phi treatment enhanced the two cytokines vs. the TBI group. However, GW9662 treatment reduced VEGFA and EGF (compared with the TBI+Phi group, Figures 7F,G). Moreover, we performed immunofluorescence and Western blot in the brain to detect MMP3, MMP9, ZO-1, occludin, and claudin-5 expressions in the TBI lesions. TBI promoted MMP3 and MMP9 in the cerebral microvessels, and inhibited ZO-1, occludin, and claudin-5 expression. On the contrary, Phi treatment reduced MMP3 and MMP9, whereas it promoted ZO-1, occludin, and claudin-5 expression in the cerebral microvessels (Figures 7H,I). However, GW9662 administration increased MMP3 and MMP9, whereas it reduced ZO-1, occludin, and claudin-5 expression (compared with the TBI+Phi group, Figures 7H,I). Overall, Phi had prominent neuroprotective effects against TBI-mediated BBB damage *via* PPARγ.

## Discussion

**FIGURE 8:**
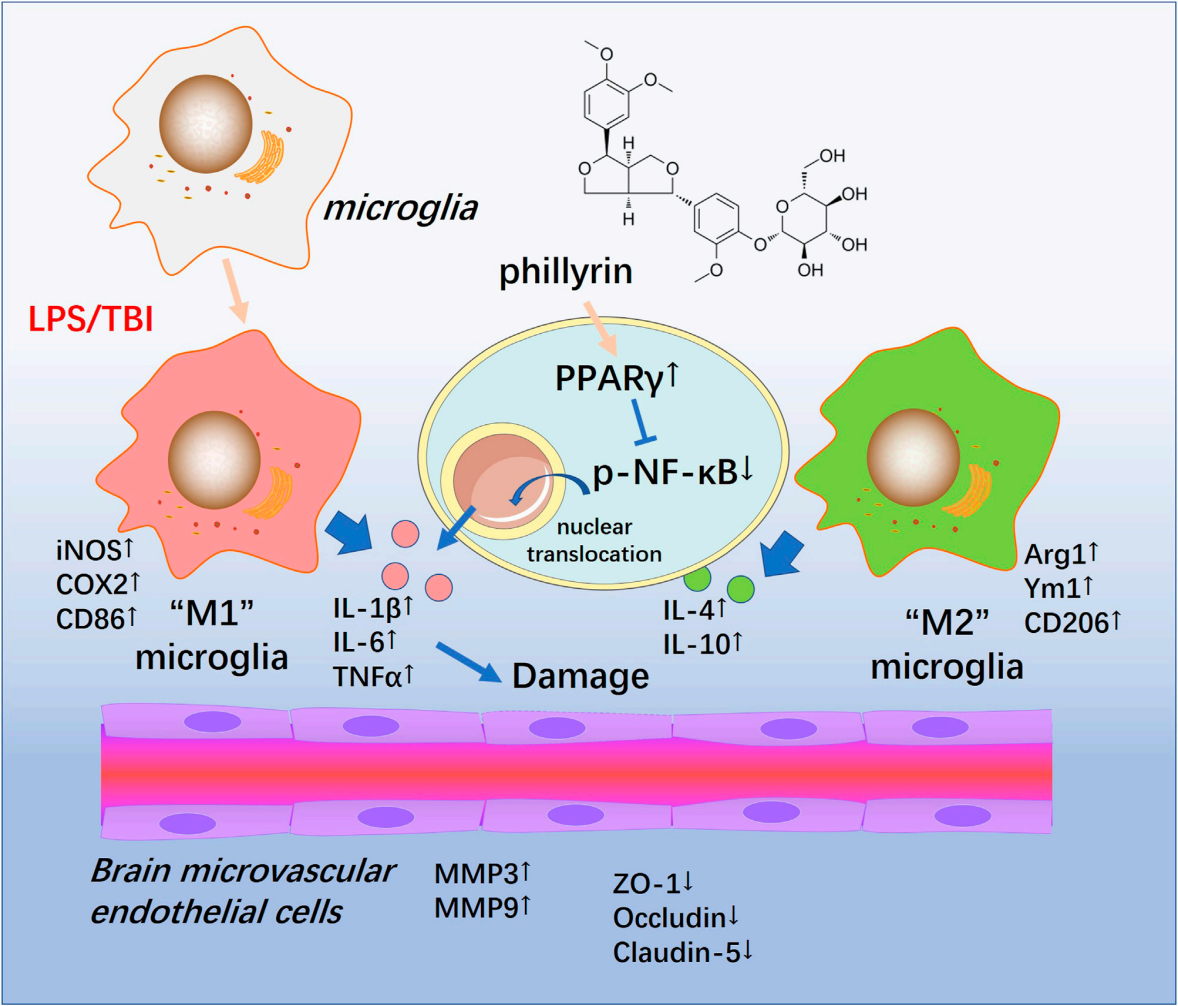
Schematic illustration of the possible mechanisms of Phi on TBI-mediated BBB damage. As illustrated, microglia become activated into an inflammatory state and release plenty of pro-inflammatory factors such as TNF-α,IL-1β, and IL-6 in the lesions of TBI. At the same time, phospho-NK-κB p65 is upregulated and translocates into the nuclei. Phi inhibits the nuclear translocation of phospho-NK-κB p65 by enhancing the PPARγ signaling pathway, promoting microglial “M2” polarization. Therefore, Phi mitigates BBB injury following TBI.

In the present study, we conducted both *in vivo* and *in vitro* experiments to evaluate the neuroprotective effects of Phi. Our data suggested that TBI activated both “M1” and "M2" polarization of microglia. Phi transformed “M1” microglia into “M2” microglia *via* modulating the PPARγ/NF-κB pathway, thereby exerting neuroprotective effects on BBB (Figure 8).

Compounds obtained from natural sources are widely being evaluated for their anti-inflammatory and antioxidative potentials in several diseases, including CNS diseases (González-Burgos and Gómez-Serranillos, 2012; Wang et al., 2020). For example, palmitoylethanolamide, enriched in soybeans, egg yolk, peanut meal, and other sources of plant and animal food, has therapeutic actions against neurodegenerative disorders, pain perception, and inflammatory diseases (Petrosino and Di Marzo, 2017). Curcumin (1,7-bis-(4-hydroxy-3-methoxyphenyl)-hepta-1,6-diene-3,5-dione), along with the other bioactive curcuminoids (demethoxycurcumin and bisdemethoxycurcumin), is found in a curry spice turmeric. *In vitro*, *in vivo* studies and clinical trials have supported that curcumin has potent anticancer, antibiotic, anti-inflammatory, and anti-aging effects (Kotha and Luthria, 2019). Tiwari et al. also showed that chronic alcohol-induced neurological deficits, neuronal apoptosis, oxidative stress, and inflammatory responses were inhibited following curcumin administration (Tiwari and Chopra, 2012; Tiwari and Chopra, 2013).

In Chinese traditional medicine, *Forsythia suspensa* (Thunb.), also known as Lianqiao, is often used for treating influenza and upper respiratory tract infection in combination with other Chinese herbal preparations (Luo et al., 2020; Zhou et al., 2017; Zhuang et al., 2020). Many bioactive ingredients isolated from *Forsythia suspensa* (Thunb.) fruits or leaves have antioxidative and anti-inflammatory effects (Xia et al., 2011). For example, the production of inflammatory mediators such as TNF-α, IL-1β, NO, and PGE2 as well as NF-κB pathway activation induced by LPS were inhibited by forsythiaside A (Wang et al., 2016). An *in vitro* study suggests that forsythiaside A exhibits therapeutic potential in Alzheimer’s disease (AD) by increasing the levels of 2-arachidonoylglycerol (2-AG) (Chen et al., 2019). Rutin, another ingredient in *Forsythia suspensa*, has been found to reduce infarct size and mitigate neuron loss in ovariectomized (OVX) rats subjected to cerebral ischemia–reperfusion (I/R) injury (Liu et al., 2018). Therefore, those ingredients from *Forsythia suspensa* (Thunb.) have potential effects in treating central nervous system diseases.

As an essential chemical composition of *Forsythia suspensa* (Thunb.), Phi has been found to express multiple biological functions, such as improving insulin resistance (Xu et al., 2019), modulating cell apoptosis, and oxidative stress response (Du et al., 2020). Surprisingly, Phi also exerts antiviral and anti-inflammatory activities against novel coronavirus (SARS-CoV-2) and human coronavirus 229E (HCoV-229E). The study conducted by Ma et al. suggested that Phi inhibits SARS-CoV-2 and HCoV-229E replication in Vero E6 cells. Moreover, Phi mitigated pro-inflammatory cytokine (TNF-α, IL-6, IL-1β, MCP-1, and IP-10) expression and repressed the NF-κB pathway in Huh-7 cells (Ma et al., 2020), which had no conflict with the preceding study (Zhong et al., 2020). Interestingly, the protective effects exerted by Phi are found in neuronal cells. Phi restrained H_2_O_2_ exposure–induced oxidative stress in PC12 cells (Wei et al., 2014). Most recently, intraperitoneal injection of Phi ameliorates neurological deficits and lesion volume by improved apoptosis and oxidative stress in intracerebral hemorrhagic mice by activating the Nrf2/HO-1 pathway (Guo et al., 2021). Our previous study had confirmed the neuroprotective effects of Phi against TBI (Jiang et al., 2020). Presently, we continue to explore the role of Phi on microglial reactions after TBI. We found that Phi inhibited microglia-mediated inflammation *via* promoting the “M2” polarization of microglia. Hence, Phi has promising therapeutic effects in TBI.

PPARγ belongs to a ligand-activated transcription factor that modulates the genes essential to various metabolic processes and cell differentiation. Recent studies have revealed that PPARγ exerts anti-inflammatory functions and therefore improves brain injury or neurodegenerative diseases (Villapol, 2018). Functionally, PPARγ is able to suppress other transcription factors, such as the transcription factor activator protein-1, Stat 1, and NF-κB. Then PPARγ inactivates macrophages (Ricote et al., 1998). PPARγ downregulates COX2, MMP9, and iNOS, indicating that PPARγ has a potential role in chronic inflammation (Lenglet et al., 2015). Several PPARγ agonists have been found to inhibit an inflammatory response of microglia/macrophage. For instance, rosiglitazone markedly attenuates middle cerebral artery occlusion–mediated brain tissue loss and white matter injury *via* reducing the number of Iba1(+)/CD16(+) M1 microglia and increasing the number of Iba1(+)/CD206(+) M2 microglia after stroke (Han et al., 2015). Interestingly, our data also showed that Phi promoted PPARγ both in microglia and TBI brain lesions of mice. Antagonizing PPARγ by GW9662 reversed Phi-mediated anti-inflammatory effects and promoted the nuclear translocation of p-NF-κB p65. Therefore, we believed that Phi exerts its effects on the “M2” polarization of microglia following TBI *via* the PPARγ/NF-κB pathway.

The blood–brain barrier (BBB) often causes damage and leakage after TBI, resulting in increased extravasation of immune cells and enhanced secondary injury (Pearn et al., 2017). Recently, increasing studies have suggested that reactive astrocytes, microglia, and monocytes are associated with BBB dysfunction and impaired homeostasis (Glushakova et al., 2018; van Vliet et al., 2020). Ameliorating overproduced inflammatory mediators from microglia helps in the repair of BBB and the improvement of long-lasting brain damage following TBI (Shi et al., 2015). In this study, we found that TBI resulted in significant BBB injury, indicated by obvious structural damage and increased caspase-3 expression of cerebral microvessels, upregulated MMP3 and MMP9, as well as downregulated tight junction proteins (including ZO-1, occludin, and claudin-5) in the cerebral microvessels. With the treatment of Phi, BBB damage caused by TBI was significantly alleviated. In the *in vitro* experiment, we utilized the condition medium of LPS-mediated microglia to treat BMECs. We found that LPS-CM inhibited cell viability and tube formation ability of BMECs, which were reversed by Phi treatment. However, those effects were all partly abolished by GW9662. Hence, we confirmed that Phi improves BBB damage following TBI by repressing inflammatory responses from microglia.

## Conclusion

In summary, the main findings of this study are that Phi has anti-inflammatory effects *via* promoting the microglial “M2” polarization through the PPARγ/NF-κB pathway, and Phi relieves TBI-induced BBB injury caused by microglia. Further research work exploring how Phi modulates the PPARγ expression in the mouse TBI brain lesions or microglia is needed, which will help to delineate more clearly the potential clinical usage of Phi.

## Data Availability

The original contributions presented in the study are included in the article/Supplementary Material; further inquiries can be directed to the corresponding author.

## Abbreviations

BBB, blood–brain barrier; BDNF, brain-derived neurotrophic factor; bFGF, basic fibroblast growth factor; BMECs, brain microvascular endothelial cells; BSA, bovine serum albumin; COX2, cytochrome c oxidase subunit I; DAMP, damage-associated molecular pattern; EGF, epidermal growth factor; ELISA, enzyme-linked immunosorbent assay; IL, interleukin; iNOS, inducible nitric oxide synthase; MMP3, matrix metallopeptidase 3MyD88, myeloid differentiation factor 88; NF-κB, nuclear factor-kappa B; PPARγ, peroxisome proliferator–activated receptor gamma; TBI, traumatic brain injury; TGF-β, transforming growth factor-β; TLR, toll-like receptor; TNF-α, tumor necrosis factor-alpha; VEGF, vascular endothelial growth factor.
